# Teachers’ competence: How to protect teachers’ mental health from the burden caused by students’ private in-class use of technical devices?

**DOI:** 10.1371/journal.pone.0305114

**Published:** 2024-06-11

**Authors:** Julia Brailovskaia, Anna-Lena Duscha, Greta M. Kreyelkamp, Jürgen Margraf

**Affiliations:** 1 Mental Health Research and Treatment Center, Department of Clinical Psychology and Psychotherapy, Ruhr-Universität Bochum, Bochum, Germany; 2 DZPG (German Center for Mental Health), Partner Site Bochum, Marburg, Germany; UN Mehta Institute of Cardiology and Research Center, INDIA

## Abstract

The current study investigated how students’ private in-class use of technical devices is associated with teachers’ mental health. Data of 361 teachers from primary and secondary schools in Germany were assessed via online surveys. The present cross-sectional results show a positive association between burden caused by the students’ private in-class use of technical devices and teachers’ depression symptoms. Both were negatively linked to teachers’ positive mental health (PMH) and teachers’ competence in handling students’ private in-class use of technical devices. In a moderated mediation analysis, the association between burden and depression symptoms was mediated by PMH. Teachers’ competence moderated the link between PMH and depression symptoms. Specifically, the higher the competence, the weaker the relationship between both variables. Thus, the protective effect of teachers’ competence could be especially important in persons with low PMH. Competence training in handling students’ use of technical devices is discussed as a potential step that could protect teachers’ mental health.

## Introduction

Digitalization belongs to the main characteristics of the 21^st^ century. It represents the involvement of new technologies in various areas of everyday life. Technical devices such as smartphones, laptops and tablets that allow their users to be always accessible and up-to-date via mobile Internet access belong to such technologies [1, 2].

For the late Generation Z (born: 1996/7 to 2010/12) and the Generation Alpha (born: 2010/12 and later), who were born after the turn of the new century, use of the new technologies often starts very early in life [3, 4]. Many members of the young generations own a smartphone when they start primary school, or when they enter secondary school at the latest [5]. Typically, they take the smartphone to school and often continue its use during classes.

However, studies focusing on young students have shed light on potential negative consequences of an intensive use of technical devices. For instance, previous research has highlighted a positive relationship between smartphone use, overweight and diminished fitness in young people [6]. In the longer-term, intensive use of technical devices can contribute to a decline of students’ physical and mental health [7]. In addition, available studies described that intensive use of technical devices such as smartphones is positively associated with a decline of young students’ academic performance (e.g., [8, 9]), a reduced attention span, and an increase in procrastination [10]. Those changes can have far-reaching consequences because students’ attention and academic performance influence their teachers’ mental health [11]. Thus, it can be assumed that students’ private in-class use of technical devices–for example uploading private content on social media such as Instagram and TikTok or writing of private WhatsApp messages via smartphones [12, 13]–not only impacts the students’ learning process, but also could burden their teachers and reduce their teachers’ mental health.

This is cause of concern considering the significance of teachers’ mental health for the education system (e.g., [14]). Notably school teachers are often exposed to a lot of stressors at their workplace [15]. As a consequence, they have an enhanced prevalence for low levels of mental health [16]. A negative climate at the classroom, students’ attention deficits, tardiness, and absenteeism can impact teachers’ mental health negatively [11, 17, 18]. Low levels of mental health can decrease the teachers’ job motivation and foster the risk for a burnout [19] which can result in a leave of the profession [15, 20]. Furthermore, teachers’ mental health is positively associated with the students’ mental health [21]. Teachers with a low level of mental health are often unable to build up and to maintain a satisfying teacher-student relationship. However, a satisfying teacher-student relationship is an important predictor of students’ learning process and academic growth [15]. In the longer-term, a poor teacher-student relationship predicts higher levels of psychiatric disorders in students [14].

**Fig 1:**
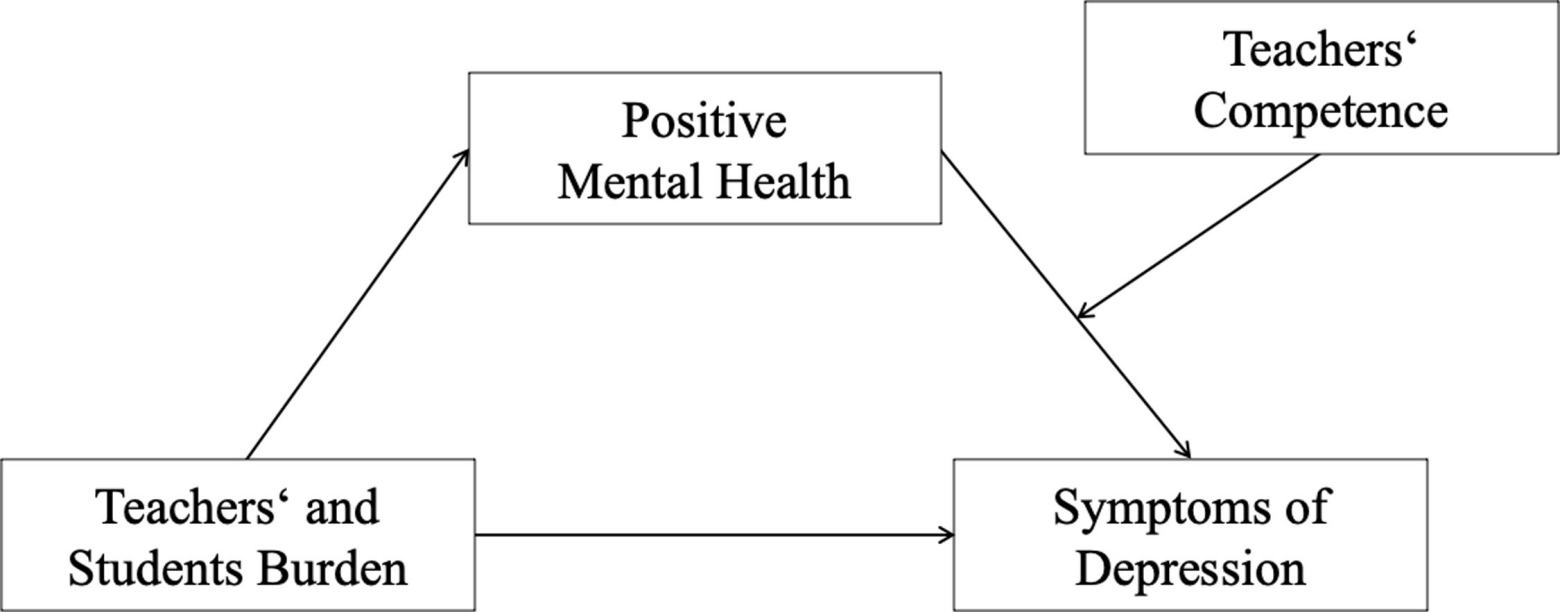
Moderated mediation model with burden experienced by students and teachers caused by students’ private in-class use of technical devices (predictor), positive mental health (mediator), teachers’ competence in handling students’ private in-class use of technical devices (moderator), and depression symptoms (outcome).

Following dual-factor models (e.g., [22]), mental health is described by two separate but interrelated dimensions: positive and negative. Persons with a high level of mental health are characterized by a low level at the negative dimension and a high level at the positive dimension [23]. Depression symptoms belong to the negative dimension of mental health [24]. Positive mental health (PMH)–that is emotional, social, and psychological well-being [25]–represents the positive dimension [24]. Considering the available literature [8, 9], we may hypothesize that students’ private in-class use of technical devices could contribute to the teachers’ depression symptoms and reduce their level of PMH. Of note, PMH confers resilience and contributes to functional coping strategies in exhausting situations [26]. It is a well-known protective factor against psychological distress that can reduce depression symptoms [26, 27]. Thus, a decrease of PMH by burden caused by students’ in-class use of technical devices could foster teachers’ depression symptoms. Specifically, PMH could serve as a buffering mediator between burden and depression symptoms (see Fig 1), and a reduction of this buffering effect could enhance depression symptoms.

Against the presented background, the aim of the current study was to investigate factors and mechanisms that could contribute to the protection of teachers’ mental health. This is of specific importance considering teachers’ enhanced prevalence for low mental health [16], their significant role for the education system [14], and the school teacher shortage in countries such as Germany [28].

Previous research described the concept of teachers’ classroom management self-efficacy [20, 29]. It indicates a teacher’s capability to gain and maintain students’ attention, and to deal with students’ misbehavior and other forms of disruptions during classes [30]. A high level of teachers’ classroom management self-efficacy can serve as a protective factor of teachers’ mental health that reduces the risk for burnout [20]. Moreover, previous research described that teachers’ general efficacy and competence in daily classes is positively associated with students’ learning efforts [31] which can predict teachers’ depression symptoms [11]. Nowadays, teachers’ classroom management self-efficacy should also include the competence to adequately handling students’ private in-class use of technical devices. Teachers should know how to control students’ private in-class use of technical devices during the lessons and have a high level of self-efficacy to implement their knowledge in daily classes. Thus, teachers’ competence in handling students’ private in-class use of technical devices could be considered as a subtype of teachers’ classroom management self-efficacy in modern classes. And it could be one of the factors that contribute to the protection of teachers’ mental health which we aimed to look for in the current study. More specifically, it might be that teachers’ competence in handling students’ private in-class use of technical devices could buffer the negative effect of reduced PMH on depression symptoms as a moderator (see Fig 1).

Thus, we aimed to investigate the relationship between burden experienced by students and teachers caused by students’ private in-class use of technical devices, teachers’ mental health (depression symptoms, PMH), and teachers’ competence in handling students’ private in-class use of technical devices. Notably, burden experienced by students such as a decrease of their academic performance is also an indirect burden for their teachers [11]. Considering the presented empirical background, we expected a positive association between burden and depression symptoms (Hypothesis 1a). In contrast, we assumed burden to be negatively linked to PMH (Hypothesis 1b) and to competence (Hypothesis 1c). Also, we assumed that depression symptoms are negatively related to PMH (Hypothesis 2a) and competence (Hypothesis 2b). And we hypothesized a positive link between PMH and competence (Hypothesis 3). Moreover, we expected that PMH could mediate the positive relationship between burden and depression symptoms (Hypothesis 4a). And we hypothesized that competence could moderate the negative link between PMH and depression symptoms (Hypothesis 4b). Specifically, the higher the level of teachers’ competence in handling students’ private in-class use of technical devices, the weaker the link between PMH and depression symptoms. Fig 1 illustrates the hypothesized relationships as a moderated mediation model [32].

## Materials and methods

### Procedure and participants

The sample consisted of 361 teachers (75.1% women, 24.9% men; age: *M* = 41.90, *SD* = 11.71, range: 24–67) from primary (35.7%) and secondary (64.3%) schools in Germany. Of them, 62.9% were married, 23.5% were in a romantic relationship, and 13.6% were singles; 58.2% were parents of at least one child. On average, the participants had about 13 years (*SD* = 10 years; range: 6 months to 42 years) teaching experience. Data were collected between May 2021 and June 2022 via an online survey. The data collection was anonymous. Thus, we had no access to information that could identify individual participants during or after data collection. We used the contact data that are free available at the online education portal of the Federal Ministry of Education of North Rhine-Westphalia (NRW) to inform the management of various primary and secondary schools about the investigation via e-mail or phone call. If the school management agreed, we sent flyers including invitations for participation and the link to the online survey via e-mail or we visited the schools to bring printed flyers and to explain the planed study in person. The school management distributed the flyers to the teaching staff. Furthermore, the flyer was uploaded in discussion groups that focused on teaching on social media such as Facebook, Twitter, and LinkedIn. Participation was voluntary and not compensated. The only requirement for participation was to be a teacher at a primary or secondary school in Germany for at least three months. There were no missing data in the completed survey. No data sets were excluded. All participants were properly instructed and gave informed consent to participate via an online form. The responsible Ethics Committee approved the implementation of the study (approval number: 705). The study has been carried out in accordance with the Declaration of Helsinki. The dataset used in the present study is available in S1 Dataset.

### Measures

We formulated the items of the burden and the competence scale for the present investigation. Before the current data collection, we implemented an expert review of the items by two trained school psychologists to enhance their content validity. In the instructions, technical devices were defined as smartphone, laptop, and tablet; private use was defined as for example writing of WhatsApp messages, uploading of content on social media such as Instagram, TikTok, and Facebook. Table 1 shows all burden and competence items.

**Table 1:** Descriptive statistics of the investigated variables, including items of burden and competence.

		*Percent*				
	*M (SD*, *Min–Max)*	“1”	“2”	“3”	“4”	“5”
Positive Mental Health	19.43 (5.91, 0–27)					
Depression Symptoms	4.01 (4.83, 0–21)					
Burden by students’ private in-class use of technical devices	21.23 (6.04, 7–35)					
*Burden Item 1*: *“Students’ private in-class use of technical devices interferes their learning process”*	3.46 (1.18, 1–5)	9.1	9.7	27.1	33.8	20.2
*Burden Item 2*: *“I am burdened by students’ private in-class use of technical devices”*	2.79 (1.29, 1–5)	19.7	24.1	25.8	18.6	11.9
*Burden Item 3*: *“I have difficulties in teaching due to students’ private in-class use of technical devices”*	2.44 (1.30, 1–5)	30.5	26.3	21.6	11.9	9.7
*Burden Item 4*: *“Students are often distracted from the learning content by their private in-class use of technical devices”*	3.08 (1.29, 1–5)	14.1	20.8	23.3	26.3	15.5
*Burden Item 5*: *“Students’ attention span is reduced by their private in-class use of technical devices”*	3.48 (1.21, 1–5)	8.0	15.0	19.7	35.7	21.6
*Burden Item 6*: *“Students’ academic performance is diminished by their private in-class use of technical devices”*	2.97 (1.21, 1–5)	13.3	21.3	33.2	19.4	12.7
*Burden Item 7*: *“Students’ private in-class use of technical devices is often discussed as a problem among the teaching staff”*	3.00 (1.28, 1–5)	15.5	20.8	26.0	23.3	14.4
Teachers’ competence in handling students private in-class use of technical devices	13.00 (3.73, 4–20)					
*Competence Item 1*: *“I was trained how to handle students’ private in-class use of technical devices”*	2.57 (1.33, 1–5)	30.5	20.5	17.5	24.4	7.2
*Competence Item 2*: *“I know how to control students’ private in-class use of technical devices”*	3.08 (1.30, 1–5)	15.2	21.1	18.0	32.1	13.6
*Competence Item 3*: *“I feel competent enough to handle students’ private in-class use of technical devices”*	3.36 (1.21, 1–5)	9.4	17.2	17,7	39.6	16.1
*Competence Item 4*: *“Students follow my advice when I ask them to stop their private in-class use of technical devices”*	3.99 (1.09, 1–5)	3.9	8.0	12.2	36.8	39.1

*N* = 361; *M* = Mean, *SD* = Standard Deviation, *Min* = Minimum, *Max* = Maximum; “1” = *strong disagreement*, “2” = *disagreement*, “3” = *neither agreement nor disagreement*, “4” = *agreement*, “5” = *strong agreement*; due to rounding, sums of the percentages are not always 100.

#### Burden by students’ private in-class use of technical devices

Burden that students and teachers experience by the students’ private in-class use of technical devices was measured by seven items (e.g., “I am burdened by students’ private in-class use of technical devices”; see Table 1). Hereby, Item 1, 4, 5, and 6 focused on the evaluation of burden experienced by students; Item 2, 3, and 7 focused on the evaluation of burden experienced by teachers. The items are rated on a 5-point Likert-type scale (1 = *strong disagreement*, 5 = *strong agreement*). The higher the sum score, the higher the burden. Current scale reliability: Cronbach’s *α* = .816.

#### Teachers’ competence in handling students’ private in-class use of technical devices

Competence of the teachers in handling students’ private in-class use of technical devices was assessed by four items (e.g., “I know how to control students’ private in-class use of technical devices”; see Table 1). The items are rated on a 5-point Likert-type scale (1 = *strong disagreement*, 5 = *strong agreement*). Higher sum scores indicate higher competence. Current scale reliability: *α* = .746.

#### Positive mental health (PMH)

We assessed PMH by the unidimensional Positive Mental Health Scale (PMH-Scale; original German language version: [25]). Available literature described the validity of the PMH-Scale across samples from various countries [33]. The nine items are rated on a 4-point Likert-type scale (e.g., “I enjoy my life”; 0 = *do not agree*, 3 = *agree*). Higher sum scores indicate higher levels of PMH. Current scale reliability: *α* = .945.

#### Depression symptoms

The depression subscales of the Depression Anxiety Stress Scales 21 (DASS-21; original version: [34]; German language version: [35]) measured depression symptoms. The DASS-21 is an internationally well-established and validated instrument for the assessment of the negative symptoms in healthy and clinical populations [36]. The seven items (e.g., “I couldn’t seem to experience any positive feeling at all”) are rated on a 4-point Likert-type scale (0 = *did not apply to me at all*, 3 = *applies to me very much or most of the time*). The higher the sum score, the higher the depression symptoms. Current scale reliability: *α* = .924.

### Statistical analyses

Statistical analyses were conducted using SPSS 28 [37] and the macro Process version 4.0 [32]. After descriptive analyses, we calculated zero-order bivariate correlations to assess the relationship between burden, competence, PMH, and depression symptoms. Then, we ran a moderated mediation analysis (Process: model 14) after ensuring that the assumptions required for this analysis (see [38]) were met. The assumptions involve the independence of observations that we tested by the Durbin-Watson statistic which provided an adequate value of 1.918 (acceptable range: 1.5 to 2.5, see [39]); linearity of relationships among the predictors and the outcome as well as the homoscedasticity of the error values that we both tested by a scatter plot analysis [38]; the absence of a violation of the multicollinearity assumption that was shown by values of tolerance *>* 0.10, and variance inflation factor values *<* 10 [40]; and normal distribution of the residuals/errors in estimation that we tested by a histogram analysis and a P-P plot [32]. The investigated moderated mediation model included a conditional indirect effect (see Fig 1). It examined the multiple effects simultaneously (integration of the hypothesized mediation and moderation effects) [41, 42]. The bootstrapping procedure (10,000 samples) which provides percentile bootstrap confidence intervals (*CI* 95%) revealed the moderated mediation effect. Burden served as predictor, PMH as mediator, competence as moderator, and depression symptoms as outcome; age, gender, and teaching experience were included as covariates. Path *a* denoted the relationship between burden and PMH; path *b* denoted the link between PMH and depression symptoms; the association between burden and depression symptoms after the inclusion of PMH and competence in the model was denoted by path *c’* (the direct effect).

## Results

Table 1 shows the descriptive statistics of the investigated variables and the response rates of the burden and competence items. Overall, 54% of the teachers agreed that the students’ private in-class use of technical devices interferes their learning process, 30.5% felt burdened by the students’ private in-class use of technical devices, and 21.6% had difficulties in teaching due to the students’ private in-class use of technical devices. Furthermore, 41.8% of the teachers agreed that students are often distracted by their private in-class use of technical devices, 57.3% reported a reduction of students’ attention, 31.1% described a negative effect on students’ academic performance, and 37.7% reported that the students’ private in-class use of technical devices is often discussed as a problem among the teaching staff. Considering teachers’ competence, 31.6% of the participants were trained how to handle the students’ private in-class use of technical devices, 45.7% knew how to control it, 55.7% felt competent enough to do it, and 75.9% agreed that their students stop the private in-class use of technical devices when they ask them to do so.

The correlation analyses showed that burden was significantly negatively correlated with PMH, *r* = -.208, *p* < .001, and with competence, *r* = -.406, *p* < .001. In contrast, burden was significantly positively correlated with depression symptoms, *r* = .300, *p* < .001. Depression symptoms were significantly negatively correlated with PMH, *r* = -.764, *p* < .001, and with competence, *r* = -.382, *p* < .001. PMH was significantly positively correlated with competence, *r* = .320, *p* < .001.

**Fig 2:**
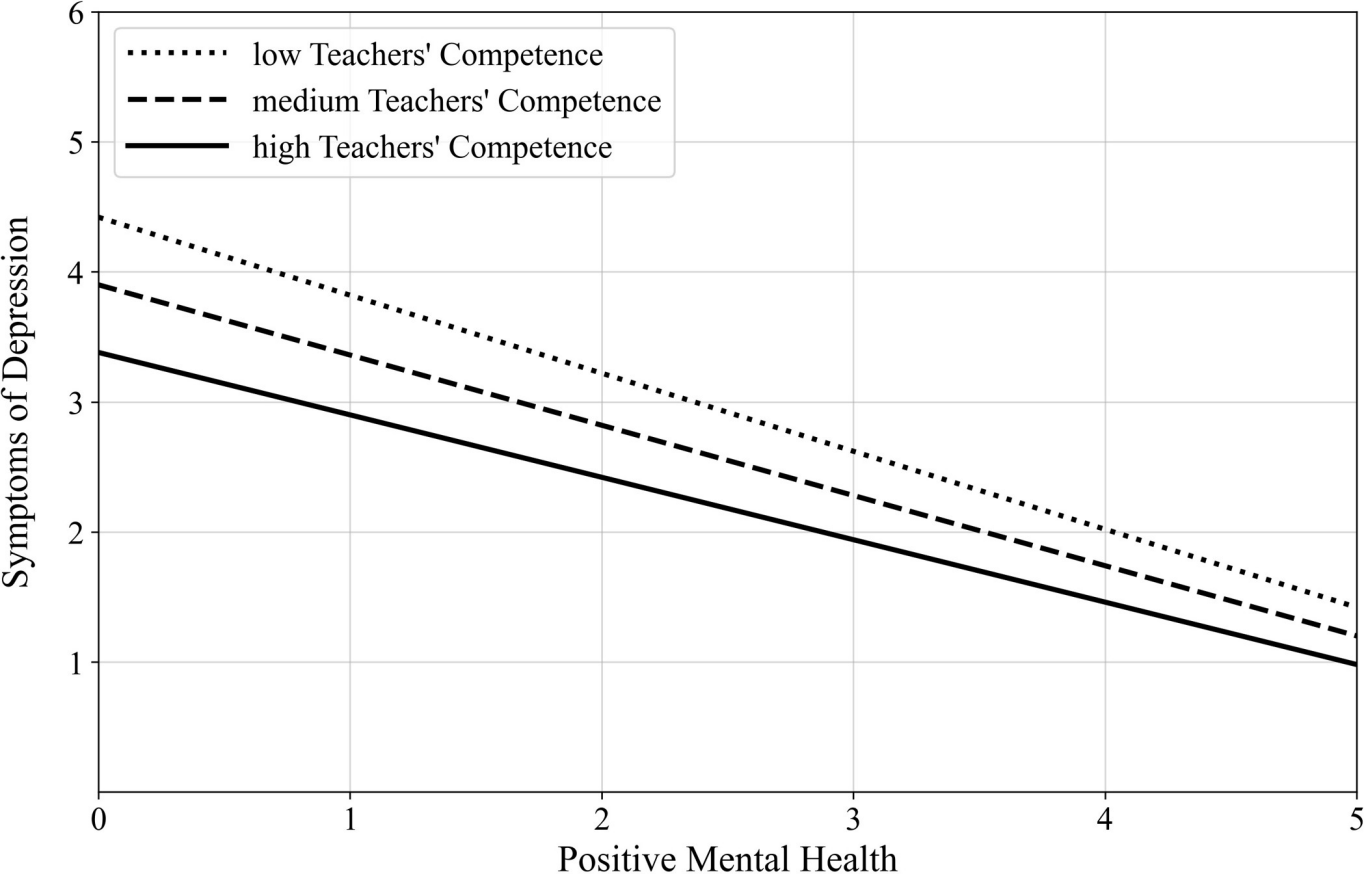
Moderation effect of teachers’ competence in handling students’ private in-class use of technical devices (moderator) on the relationship between positive mental health (predictor) and depression symptoms (outcome).

The moderated mediation analysis revealed a significant overall model, *F*(7,353) = 86.587, *p* < .001 (see Table 2). The explained variance of the overall model was *R*^*2*^ = .632. The direct effect (path *c’*) of burden on depression symptoms was significant (*p* = .019) after controlling for PMH, competence, and their interaction. The conditional indirect effect of burden on depression symptoms through PMH was significant in teachers with low (that is one *SD* below the mean = -3.726), medium (that is the mean = 0) and high (that is one *SD* above the mean = 3.726) levels of competence. But the higher was the competence, the lower was the effect (low > medium > high competence; see Fig 2). The index of moderated mediation indicated that the test of moderated mediation was significant. Thus, there was a significant moderated mediation effect (see Table 2).

**Table 2:** Moderated mediation model (outcome: Depression symptoms).

	ß	SE	t	*p*	95% *CI*
Path *a*: Burden → PMH	-.173	.051	-3.371	< .001	[-2.741, -.072]
Path *b*: PMH → Depression Symptoms	-.543	.030	-18.381	< .001	[-.601, -.485]
Interaction: PMH*Competence → Depression Symptoms	.016	.007	2.361	.019	[.003, .030]
Path *c’* (direct effect): Burden → Depression Symptoms	.069	.029	2.362	.019	[.012, .126]
*Conditional Indirect Effects*: *Burden* → *Depression Symptoms*					
Burden → PMH → Depression Symptoms					
Competence:					
Low (one *SD* below mean = -3.726)	.105	.039			[.033, .184]
Medium (mean = 0)	.094	.034			[.030, .164]
High (one *SD* above mean = 3.726)	.083	.030			[.027, .147]
*Index of Moderated Mediation*	-.003	.002			[-.008, -.001]

*N* = 361; covariates: age, gender, teaching experience; PMH = Positive Mental Health; burden = burden experienced by students and teachers caused by students’ private in-class use of technical devices; competence = teachers’ competence in handling students’ private in-class use of technical devices; ß = Standardized Beta; SE = Standard Error; *t* = *t*-test; *p* = significance; *CI* = Confidence Interval; explained variance of the overall model: *R*^*2*^ = .632.

## Discussion

The use of technical devices starts very early in life of the late Generation Z and the Generation Alpha (e.g., [4]). Most of them own a smartphone and use it intensively over the course of a day at school and during leisure time [5]. Previous research showed that intensive engagement in the use of technical devices is associated with the young people mental health [9, 43, 44]. The present findings extend this knowledge. They show that the use is also linked to the mental health of their teachers. Moreover, they reveal potential factors and mechanisms that could protect the teachers’ mental health in the age of the digital revolution.

Earlier studies emphasized that teachers are at enhanced risk for low levels of mental health compared to the general population [16, 45]. Our results show that nowadays students’ private in-class use of technical devices could be one of the reasons for this finding. The teachers’ responses revealed that students’ private in-class use of technical devices in primary and secondary schools in Germany can burden the students. It can interfere and distract their learning process, reduce their attention and academic performance. Furthermore, it can burden the teachers and cause difficulties for the teaching process which is a problem that is often discussed among the teaching staff.

Available research described that students’ attention deficits can negatively impact the climate in the classroom which burdens the teachers and decreases their mental health [11]. In line with these findings, burden by students’ private in-class use of technical devices was positively associated with teachers’ depression symptoms (confirmation of Hypothesis 1a). Its relationship with teachers’ PMH was negative (confirmation of Hypothesis 1b). In addition, teachers’ PMH and teachers’ depression symptoms were negatively associated (confirmation of Hypothesis 2a). Moreover, teachers’ PMH mediated the positive link between burden by students’ private in-class use of technical devices and teachers’ depression symptoms (confirmation of Hypothesis 4a). PMH is an important protective factor that fosters one’s resilience and functional coping strategies in stressful situations [26]. High levels of PMH can reduce the risk of depression symptoms and other mental problems such as anxiety disorders [46, 47]. Following our findings, burden by students’ private in-class use of technical devices could reduce teachers’ PMH. Less PMH implies a reduced protection effect and thus a higher risk for mental problems [48]. Of note, teachers’ depression symptoms can impact students’ mental health and their academic performance [21]. Furthermore, they can foster the risk for teachers’ burnout which can result in a temporary absence from work or a total leave of the profession [15, 20].

Against this background, it is of great importance to identify and to promote further protective factors that could compensate for low levels of PMH. The current findings reveal teachers’ competence in handling students’ private in-class use of technical devices–that we suggest to consider as a subtype of teachers’ classroom management self-efficacy [20]–as one of such protective factors. Teachers’ competence was negatively associated with burden by students’ private in-class use of technical devices (confirmation of Hypothesis 1c) and with teachers’ depression symptoms (confirmation of Hypothesis 2b). The association between teachers’ competence and teachers’ PMH was positive (confirmation of Hypothesis 3). Furthermore, teachers’ competence moderated the link between teachers’ PMH and teachers’ depression symptoms (confirmation of Hypothesis 4b). Specifically, the higher the level of the competence, the weaker the association between both variables. This means that in teachers with a high competence level the decrease of PMH contributes less to an increase of depression symptoms. In contrast, teachers with a low competence level are at enhanced risk for depression symptoms when their PMH level decreases.

The use of technical devices is an essential part of young students’ everyday life [9]. Often, they are not aware of the potential negative consequences of their permanent use for themselves and for other people. Therefore, it is the duty of adults who surround them–parents and teachers–to control their use, to explain the potential consequences of the use and to teach them how to handle the use competently. At school, teachers should, on the one hand, know how to incorporate technical devices for meaningful learning [49]. On the other hand, our findings show that they should be also able to control students’ private in-class use of technical devices. This can protect teachers’ mental health which can have a positive impact on the climate in the classroom, the students’ mental health, support their academic growth and performance [21]. As a consequence, the level of teachers’ stress and the risk for a burnout can decrease [15]. Thus, a training of teachers’ competence in handling students’ private in-class use of technical devices is an essential step that can protect students’ and teachers’ mental health as well as the students’ performance in the 21^st^ century. Considering the results of the moderated mediation model, especially teachers with low levels of PMH who are at risk for high depression symptoms could benefit from the competence training. This training could contribute to the teachers’ general ability to manage their classroom that is of great importance to achieve positive educational outcomes [50]. And as a consequence, their PMH level which is positively associated with the competence in handling students’ private in-class use of technical devices could also be increased.

The present findings emphasize that there is a significant need for such a competence training. Less than one third of our participants has been previously trained how to handle their students’ private in-class use of technical devices and less than a half of them knew how to control the use. Thus, there were a lot of teachers who did not know how to handle the students’ private in-class use of technical devices or who did not feel competent enough to transfer their knowledge–if available–in practice in their classroom.

Against this background, to protect teachers’ mental health and to reduce the teacher shortage, federal governments and authorities as well as school management should focus on the improvement of teachers’ competence in handling students’ private in-class use of technical devices. Available research showed that education trainings for teachers’ that focus on their class management abilities often include didactic instructions, coaching, and performance feedback [50]. Considering our findings, it seems reasonable that in such expert-guided trainings, teachers could also be educated in the impact of the use of technical devices on various areas of life including mental and physical health, in what functional and dysfunctional use of technical devices is, and in how to manage and control the use of their students. Furthermore, the improvement of teachers’ PMH should be focused. Following previous research [51, 52], PMH can be fostered by a training of mindfulness–the ability to maintain the focus of attention in the present moment [53], as well as by physical activity such as jogging, swimming and cycling that reduces symptoms of stress and provides positive emotions [54, 55]. Especially physical activity in a group setting can enhance one’s feeling of social support [56] that is an important predictor of PMH [57]. Thus, teachers should be offered low-threshold opportunities to participate in mindfulness-training, group training in physical activity and competence training on handling students’ private in-class use of technical devices. In the longer-term, this could have a positive impact on the general climate at schools and students’ achievements and mental health.

The following limitation should be considered when interpreting present findings. First, the cross-sectional study design allows only hypothetical conclusions on causality. For true causal conclusions, our findings should be replicated by experimental research with several measurement time points. For example, teachers’ competence in handling students’ private in-class use of technical devices could be enhanced by specific training programs. After the training, it could be investigated whether the increased competence contributes to a decrease of teachers’ depression symptoms and whether persons with low PMH levels especially benefit from this intervention immediately after it, three and six months later. Second, the present self-selected sample was relatively small and included mostly female teachers from Germany. Notably, the German Federal Statistical Office reported that about 73% of the teachers in Germany were female in the academic year 2020/2021 [58]. Therefore, our sample was relatively similar to teacher demographics in Germany considering gender. Nevertheless, a larger sample with a higher male proportion is desirable for future studies that aim to replicate our findings. Moreover, we used only online surveys for data collection which could result in the exclusion of teachers who do not use the Internet. This limits the generalizability of the present findings to the general national and international teacher population. To assess the findings’ representativeness, the present results could be replicated in larger and more gender-balanced groups in various countries. Furthermore, data should be assessed not only online but also via pencil-and-paper surveys. In addition, due to the voluntary nature of study participation, we cannot exclude a participation bias. Specifically, it could be that our offline and online distributed flyers called especially the attention of teachers who felt overwhelmed by the students’ private in-class use of technical devices. Third, we included only self-report instruments that are prone to same-source bias and social desirability [59, 60]. Thus, we suggest to replicate our findings by the measurement of and control for social desirability in statistical analyses (e.g., Balanced Inventory of Social Desirability [59]). Fourth, we developed the burden scale and the competence scale for the current study with the support of expert reviews, which enhanced their content validity. However, a further validation of both instruments in future studies is desirable. Notably, the burden scale aims to assess the burden experienced by students and teachers due to students’ private in-class use of technical devices. While the items focusing on the burden experienced by teachers include a self-description/evaluation of the teachers, the items focusing on the burden experienced by students imply evaluations of the students by their teachers. These differing evaluation focuses (self-description and external perception) could influence the validity of this instrument. Therefore, we suggest comparing the responses of teachers and their students provided to the burden scale in future research. Furthermore, we suggested that teachers’ competence in handling students’ private in-class use of technical devices that we assessed by the 4-item competence scale could be a subtype of teachers’ classroom management self-efficacy. However, we did not assess other forms of self-efficacy in the present study that we could use for a further validation of this idea. Therefore, we recommend future research to include for example the classroom management subscale of the Ohio State Teacher Efficacy Scale (OSTES) [29] and the Generalized Self-Efficacy Scale (GSES) [61] to assess the construct validity of our competence scale. And future research should investigate the suggested role of teachers’ competence in handling students’ private in-class use of technical devices as a subtype of teachers’ classroom management self-efficacy. Fifth, we focused on students’ private in-class use of technical devices in the present study. Future studies should extend our findings by the inclusion of students’ in-class use of technical devices for meaningful learning instructed by teachers to get a full overview of the use and its associations. Sixth, we assessed only depression symptoms as mental health problems. Future studies should include further mental problems to better understand the potential impact of students’ private in-class use of technical devices. Seventh, we collected data between May 2021 and June 2022. During this period, the extent of mitigation measures, such as the need for “social distancing” and online learning at schools, introduced to fight the spread of the coronavirus disease 2019 (Covid-19) in Germany changed several times [62]. Available literature has shown that for many people, the outbreak of Covid-19 and the changing mitigation measures were associated with an enhanced level of mental strain [63]. The present study did not assess Covid-19-specific factors. Therefore, we cannot exclude the possibility that teachers who participated in our study in 2021 and 2022 were differently impacted by Covid-19 and the mitigation measures which could impact our findings.

## Conclusions

In conclusion, the current study reveals that the burden caused by students’ private in-class use of technical devices could impact their teachers’ mental health. It could reduce their level of PMH and increase their depression symptoms. Teachers’ mental health is an important predictor of students’ mental health and academic performance [15]. This emphasizes the importance to protect and to foster teachers’ mental health. The current results show that teachers’ competence in handling students’ private in-class use of technical devices is of specific significance for teachers’ mental health. However, many teachers lack this competence today. Its training that is offered and promoted by governments and school management could foster teachers’ mental health, and thus it could improve the school climate and the students’ mental health and academic achievements.

## Supporting information

S1 Dataset Dataset used for analyses in present study. (SAV)

S1 Checklist STROBE statement—checklist of items that should be included in reports of observational studies. (DOCX)
