# Supplementary material for: Teachers’ competence: How to protect teachers’ mental health from the burden caused by students’ private in-class use of technical devices?
Source: PLoS One. 2024 Jun 11;19(6):e0305114. doi: 10.1371/journal.pone.0305114 (PMC11166289; doi:10.1371/journal.pone.0305114)
Supplement: S1 Checklist — (DOCX) [file pone.0305114.s002.docx]

STROBE Statement—checklist of items that should be included in reports of observational studies

|  | Item No. | Recommendation | Page  No. | Relevant text from manuscript |
| --- | --- | --- | --- | --- |
| **Title and abstract** | 1 | (*a*) Indicate the study’s design with a commonly used term in the title or the abstract | 2 | Data of 361 teachers from primary and secondary schools in Germany were assessed via online surveys. The current cross-sectional results reveal |
|  |  | (*b*) Provide in the abstract an informative and balanced summary of what was done and what was found | 2 | See the Abstract |
| Introduction | | | |  |
| Background/rationale | 2 | Explain the scientific background and rationale for the investigation being reported | 3-6 | See Introduction section |
| Objectives | 3 | State specific objectives, including any prespecified hypotheses | 5-6 | See Introduction section |
| Methods | | | |  |
| Study design | 4 | Present key elements of study design early in the paper | 6 | Data were collected between May 2021 and June 2022 via an online survey. |
| Setting | 5 | Describe the setting, locations, and relevant dates, including periods of recruitment, exposure, follow-up, and data collection | 6-7 | See Method section |
| Participants | 6 | (*a*) *Cohort study*—Give the eligibility criteria, and the sources and methods of selection of participants. Describe methods of follow-up  *Case-control study*—Give the eligibility criteria, and the sources and methods of case ascertainment and control selection. Give the rationale for the choice of cases and controls  *Cross-sectional study*—Give the eligibility criteria, and the sources and methods of selection of participants | 6-7 | We used the contact data that are free available at the online education portal of the Federal Ministry of Education of North Rhine-Westphalia (NRW) to inform the management of various primary and secondary schools about the investigation via e-mail or phone call. If the school management agreed, we sent flyers including invitations for participation and the link to the online survey via e-mail or we visited the schools to bring printed flyers and to explain the planed study in person. The school management distributed the flyers to the teaching staff. Furthermore, the flyer was uploaded in discussion groups that focused on teaching on social media such as Facebook, Twitter, and LinkedIn. Participation was voluntary and not compensated. The only requirement for participation was to be a teacher at a primary or secondary school in Germany for at least three months. |
|  |  | (*b*) *Cohort study*—For matched studies, give matching criteria and number of exposed and unexposed  *Case-control study*—For matched studies, give matching criteria and the number of controls per case | N(A |  |
| Variables | 7 | Clearly define all outcomes, exposures, predictors, potential confounders, and effect modifiers. Give diagnostic criteria, if applicable | 7-9 | See Method section |
| Data sources/ measurement | 8* | For each variable of interest, give sources of data and details of methods of assessment (measurement). Describe comparability of assessment methods if there is more than one group | *6-9* | See Method section |
| Bias | 9 | Describe any efforts to address potential sources of bias | 6-7 | See Method section |
| Study size | 10 | Explain how the study size was arrived at | 6-7 | See Method section |

Continued on next page

| Quantitative variables | 11 | Explain how quantitative variables were handled in the analyses. If applicable, describe which groupings were chosen and why | 10 | See Method section |
| --- | --- | --- | --- | --- |
| Statistical methods | 12 | (*a*) Describe all statistical methods, including those used to control for confounding | 10 | See Method section |
|  |  | (*b*) Describe any methods used to examine subgroups and interactions | 10 | See Method section |
|  |  | (*c*) Explain how missing data were addressed | 7 | There were no missing data in the completed survey. |
|  |  | (*d*) *Cohort study*—If applicable, explain how loss to follow-up was addressed  *Case-control study*—If applicable, explain how matching of cases and controls was addressed  *Cross-sectional study*—If applicable, describe analytical methods taking account of sampling strategy | N/A |  |
|  |  | (*e*) Describe any sensitivity analyses | N/A |  |
| Results | | | | |
| Participants | 13* | (a) Report numbers of individuals at each stage of study—eg numbers potentially eligible, examined for eligibility, confirmed eligible, included in the study, completing follow-up, and analysed | 10-12 | See Results section |
|  |  | (b) Give reasons for non-participation at each stage | N/A |  |
|  |  | (c) Consider use of a flow diagram | N/A |  |
| Descriptive data | 14* | (a) Give characteristics of study participants (eg demographic, clinical, social) and information on exposures and potential confounders | 6-7 | See Method section |
|  |  | (b) Indicate number of participants with missing data for each variable of interest | 7 | There were no missing data in the completed survey. |
|  |  | (c) *Cohort study*—Summarise follow-up time (eg, average and total amount) | N/A |  |
| Outcome data | 15* | *Cohort study*—Report numbers of outcome events or summary measures over time | N/A |  |
|  |  | *Case-control study—*Report numbers in each exposure category, or summary measures of exposure | N/A |  |
|  |  | *Cross-sectional study—*Report numbers of outcome events or summary measures | 10-12 | See Results section |
| Main results | 16 | (*a*) Give unadjusted estimates and, if applicable, confounder-adjusted estimates and their precision (eg, 95% confidence interval). Make clear which confounders were adjusted for and why they were included | 10-12 | See Results section |
|  |  | (*b*) Report category boundaries when continuous variables were categorized | 10-12 | See Results section |
|  |  | (*c*) If relevant, consider translating estimates of relative risk into absolute risk for a meaningful time period | N/A |  |

Continued on next page

| Other analyses | 17 | Report other analyses done—eg analyses of subgroups and interactions, and sensitivity analyses | 10-12 | See Results section |
| --- | --- | --- | --- | --- |
| Discussion | | | | |
| Key results | 18 | Summarise key results with reference to study objectives | 12-17 | See Discussion section |
| Limitations | 19 | Discuss limitations of the study, taking into account sources of potential bias or imprecision. Discuss both direction and magnitude of any potential bias | 16-17 | See Discussion section |
| Interpretation | 20 | Give a cautious overall interpretation of results considering objectives, limitations, multiplicity of analyses, results from similar studies, and other relevant evidence | 12-17 | See Discussion section |
| Generalisability | 21 | Discuss the generalisability (external validity) of the study results | 12-17 | See Discussion section |
| Other information | |  | | |
| Funding | 22 | Give the source of funding and the role of the funders for the present study and, if applicable, for the original study on which the present article is based | See Submission System |  |

*Give information separately for cases and controls in case-control studies and, if applicable, for exposed and unexposed groups in cohort and cross-sectional studies.

**Note:** An Explanation and Elaboration article discusses each checklist item and gives methodological background and published examples of transparent reporting. The STROBE checklist is best used in conjunction with this article (freely available on the Web sites of PLoS Medicine at http://www.plosmedicine.org/, Annals of Internal Medicine at http://www.annals.org/, and Epidemiology at http://www.epidem.com/). Information on the STROBE Initiative is available at www.strobe-statement.org.
